# Promoting tumorigenesis in nasopharyngeal carcinoma, NEDD8 serves as a potential theranostic target

**DOI:** 10.1038/cddis.2017.195

**Published:** 2017-06-01

**Authors:** Ping Xie, Jun-Ping Yang, Yun Cao, Li-Xia Peng, Li-Sheng Zheng, Rui Sun, Dong-Fang Meng, Meng-Yao Wang, Yan Mei, Yuan-Yuan Qiang, Li Cao, Yan-Qun Xiang, Dong-Hua Luo, Jing-Ping Yun, Bi-Jun Huang, Li-Jun Jia, Chao-Nan Qian

**Affiliations:** 1 State Key Laboratory of Oncology in South China, Collaborative Innovation Center for Cancer Medicine, Sun Yat-sen University Cancer Center, Guangzhou, Guangdong, China; 2 Department of Pathology, Sun Yat-sen University Cancer Center, Guangzhou, Guangdong, China; 3 Department of Nasopharyngeal Carcinoma, Sun Yat-sen University Cancer Center, Guangzhou, Guangdong, China; 4 Radiotherapy Department, Affiliated Cancer Hospital of Guangzhou Medical University, Guangzhou, Guangdong, China; 5 Cancer Institute, Fudan University Shanghai Cancer Center; Department of Oncology, Shanghai Medical College, Fudan University, Shanghai, China

## Abstract

Nasopharyngeal carcinoma (NPC), is one of the most common human malignancies in south China, it has the highest recurrence rate and treatment resistance. The underlying molecular mechanisms of NPC relapse and treatment tolerance are not fully understood. In this study, the effects of NEDD8 and NEDD8-activating enzyme inhibitor (MLN4924) on NPC were studied both *in vitro* and *in vivo*. Immunohistochemical staining of 197 NPC tissues revealed an elevated NEDD8 expression as an unfavorable independent factor in overall survival and disease-free survival rates. NEDD8 expression was positively correlated with a high risk of death and positivity of lymph node metastasis. Depleted NEDD8 expression by shRNA and inhibited by specific inhibitor MLN4924 dramatically suppressed cell proliferation, cell apoptosis, cell cycle arrest, while ectopic NEDD8 exhibited opposing effects. NEDD8 affected cancer stem cell phenotypes of NPC as assessed *in vitro* using the cell number of side population (SP) by flow cytometry analysis, colony formation assay, sphere formation assay, and tumor initiation ability *in vivo*. Downregulation of NEDD8 enhanced the susceptibility of NPC cells to cisplatin and radiation. Moreover, we found that MLN4924 suppressed c-Jun degradation in human NPC cells. Taken together, this report revealed that NEDD8 may act as a novel prognostic marker and MLN4924 may serve as a promising therapeutic target for patients with NPC.

Nasopharyngeal carcinoma (NPC) is an endemic malignancy with a high incidence rate in southern China and Southeast Asia.^1, 2, 3^ Although NPC is sensitive to radiotherapy and chemotherapy, distant metastasis is the primary cause of treatment failure.^4, 5^ Despite identification of several key molecules driving NPC metastasis in our previous studies,^6, 7, 8, 9, 10^ the molecular mechanisms underlying NPC progression and metastasis are not fully understood. For metastatic NPC, cisplatin-based chemotherapy has become the standard treatment. However, treatment resistance inevitably occurs in most metastatic NPCs. Therefore, the development of effective molecular targeted agents is urgently needed to prolong survival through overcoming metastasis and therapeutic resistance.

The ubiquitin–proteasome system (UPS) is a well-known post-translational protein modification process that plays an important role in the mediation of the proteasome-dependent degradation of intracellular proteins.^11^ Neddylation, a homologous pathway to ubiquitination, is the process of adding ubiquitin-like molecule NEDD8 (an 81–amino acid protein with a 9-kDa relative molecular mass) to target proteins.^12, 13^ NEDD8 is first activated by a NEDD8-activating enzyme (NAE), and is transferred to an E2 enzyme and conjugated to E3 ligases.^14^ The function of neddylation is to regulate a variety of molecular degradations through ubiquitination modification. It has been demonstrated that neddylation has an essential role in cellular survival, contributing to uncontrolled proliferation, genomic instability, and cancer.^14, 15^ Consequently, further study of NEDD8’s function and inhibition protein neddylation, have emerged as novel anticancer strategies. MLN4924 is a selective NAE inhibitor that has been reported to be a promising anticancer drug candidate.^15^ In cellular and animal models of lymphoma, colorectal cancer, liver cancer, pancreatic carcinoma, bladder urothelial carcinoma and breast cancer, MLN4924 has been shown to inhibit tumor cell proliferation and metastasis.^16, 17, 18, 19, 20, 21^ MLN4924 has also been reported to enhance p21-dependent radio-sensitization in human breast cancer cells and suppress tumor angiogenesis.^21, 22^ To date, the detailed mechanisms of NEDD8 and its inhibitor MLN4924 in human NPC remains unknown.

Cancer stem cells (CSCs), which make up a small proportion of tumor cells, have a key role in tumor initiation, recurrence and metastasis.^23, 24^ CSCs have been considered important therapeutic targets for anticancer treatments. It has been reported that small percentage of NPC cells has properties of CSCs.^25^ Our previous study also discovered the existence of CSC-like CD44+ cells in NPCs, which were responsible for the radio-resistance of NPC cells.^26^ However, it remains uncertain as to whether NEDD8 is involved in the maintenance of CSCs in NPC.

In the present study, we explored the role of NEDD8 in NPC growth, drug resistance and stemness characteristics, as well as evaluated the therapeutic efficacy of MLN4924 in NPC.

## Results

### High NEDD8 expression in primary NPC indicates poorer patient prognosis

**Figure 1:**
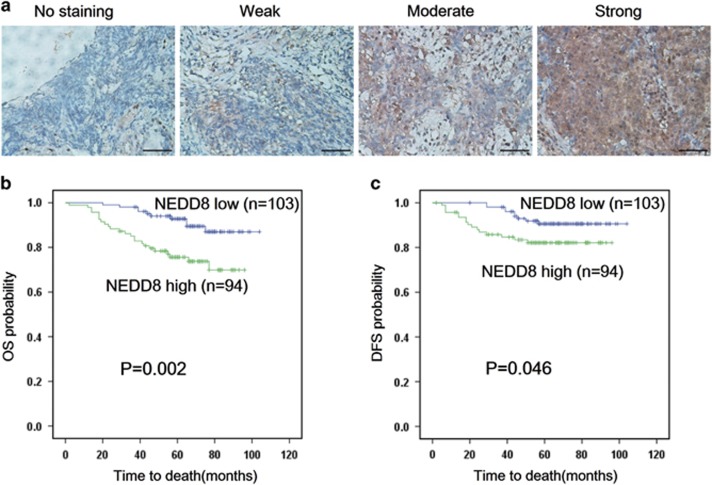
Elevated NEDD8 level correlates with shorter overall survival and disease-free survival in NPC patients. (**a**) NEDD8 protein levels in 197 NPC tissues were analyzed by IHC. The images represented the different staining intensities of the NEDD8 protein. Scale bars, 50 *μ*m. (**b,c**) The correlation between NEDD8 expression and overall survival (OS) rate (*P*=0.002) and disease-free survival rate (*P*=0.046) of NPC patients was determined using Kaplan–Meier survival and log-rank analysis

We first evaluated the prognostic value of NEDD8 expression in NPC samples. As shown in Figure 1a. IHC staining revealed that NEDD8 was localized in the cytoplasm and nucleus of NPC cells. A total of 197 patient samples were included and divided into two groups: low NEDD8 expression group (*n*=103) and high NEDD8 expression group (*n*=94). The median value of immunoreactivity scores were used as the cut-off value. The *χ*^2^ test revealed that the elevated NEDD8 expression in primary tumors were significantly correlated with a higher death risk (*P*=0.003) and a positive lymph node involvement (*P*=0.023) (Table 1). Kaplan–Meier survival curves and log-rank tests revealed that NEDD8 overexpression levels were significantly correlated with overall survival (OS) and disease-free survival (DFS) in NPC patients (Figures 1b and c). The OS and DFS were longer in patients with lower levels of NEDD8 expression, in contrast to patients with higher levels of NEDD8 expression. Multivariate analyses revealed that NEDD8 expression levels, with a hazard ratio (HR) of 0.303 and a 95% CI of 0.144–0.640, were independent prognostic factors in patients with NPC (Table 2). These analyses confirm the critical role of NEDD8 in NPC progression.

### Suppression of NEDD8 inhibits NPC cell growth *in vitro*

**Figure 2:**
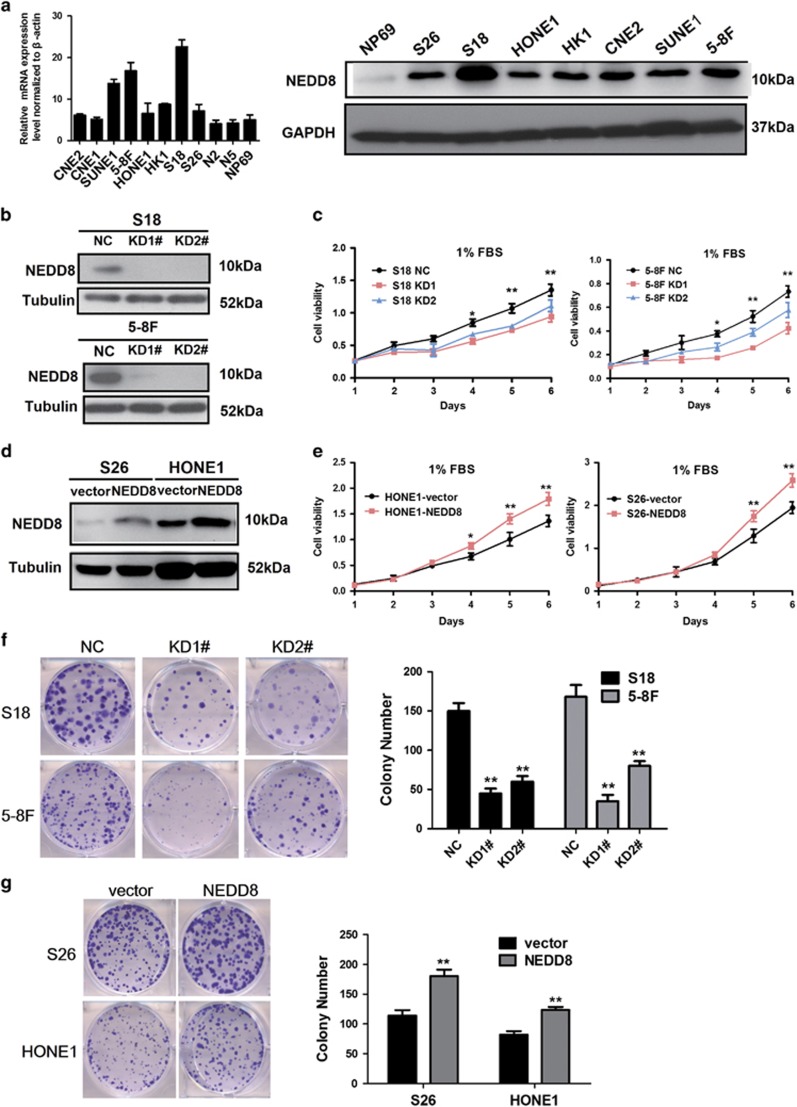
Regulation of NEDD8 expression affects growth in NPC cells *in vitro*. (**a**) NEDD8 was differentially expressed in NPC cell in mRNA and protein levels. (**b**,**d**) Stabled suppression and overexpression of NEDD8 in NPC cells were determined by immunoblotting analysis, tubulin was used as a loading control. (**c**,**e**) Cell proliferation was measured by the MTT assay with 1% FBS culture medium. (**f**,**g**) The colony formation assays were performed in the indicated stable cell lines. Three independent experiments were performed; *P<0.05, **P<0.01 *versus* vector or NC control cells

The expression of NEDD8 was detected in NPC cells using real-time quantitative PCR (RT-qPCR) and immunoblotting analyses. Increased NEDD8 mRNA and protein levels were observed in NPC cell lines and compared with N2 and N5 normal nasopharyngeal epithelial cells, as well as immortalized nasopharyngeal epithelial cells NP69 (Figure 1a). In order to explore the role of NEDD8 in NPC cell proliferation, S18 and 5-8F cells were transfected with either shRNA (NEDD8 KD1 and KD2) or a negative control lentivirus. The suppression efficiency of NEDD8 protein levels were confirmed by immunoblotting (Figure 2b). Knocking down NEDD8 significantly inhibited S18 and 5-8F cell proliferation (Figure 2c) in 1% FBS culture conditions, as well as its colony formation ability (Figure 2f), but there was no influence when cells were cultured with a 10% FBS medium (results are not provided). Meanwhile, we established NEDD8 overexpressing cell lines (S26 and HONE1). NEDD8 protein expression was validated by immunoblotting (Figure 2d). Consistently, overexpression of NEDD8 in both S26 and HONE1 cells enhanced cellular proliferation (Figure 2e) and colony formation (Figure 2g). Taken together, these results show that NEDD8 can promote NPC cell growth.

### Knockdown of NEDD8 enhances cisplatin and radiation sensitivity of NPC cells

**Figure 3:**
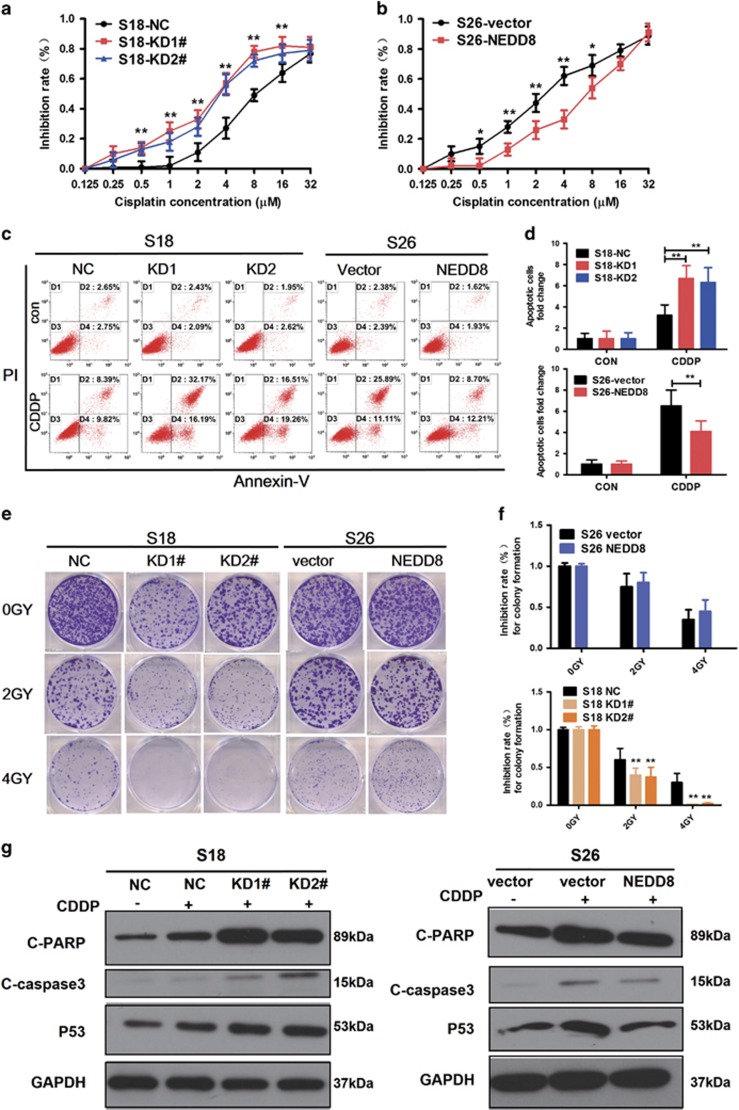
NEDD8 induces the sensitivity of cisplatin and radiation in NPC cells. (**a,b**) S18 cells with silenced NEDD8 and S26 cells with overexpressed NEDD8 were seeded in 96-well plates at a density of 1 × 10^3^ per well and treated different concentrations cisplatin (CDDP) as indicated for 72 h. Cell viabilities were tested by the MTT assay. (**c**,**d**) The cells were seeded in 6-well plates at a density of 1 × 10^5^ per well and treated with 8 *μ*M CDDP for 48 h. The apoptosis rates were detected with the Annexin-V/PI kit and analyzed by flow cytometry. (**e**,**f**) The cells were treated with different GY of radiation. Representative images (**e**) and the inhibition rate of colonies formed (**f**) are shown. (**g**) Western blotting analysis of cleaved PARP, cleaved-caspase-3 and p53. Three independent experiments were performed for each assay. The quantified results were presented as the means±S.E.M. (*n*=3). *P<0.05, **P<0.01

It was observed that S18 cells with NEDD8 knockdown displayed reduced cellular viability in the presence of varying concentrations of cisplatin, while the viability of S26 cells were enhanced by NEDD8 overexpression (Figures 3a and b). As measured by Annexin-V/PI staining, the apoptotic rates were similar in NEDD8-silencing cells (NC *versus* KD1 versus KD2) and in NEDD8-expressing cells (vector *versus* NEDD8) without cisplatin treatment. After cisplatin exposure, the apoptotic index increased more obviously in NEDD8-silencing S18 cells when compared with NC cells (Figures 3c and d). Conversely, NEDD8-expressing S26 cells appeared to have a reduced amount of apoptosis cells after cisplatin treatment (Figures 3c and d). These results were further supported by the cleavage of PARP and caspase-3, as well as the p53 protein expression (Figure 2g). The levels of cleaved PARP, cleaved caspase-3 and p53 were dramatically increased in NEDD8-silenced cells after cisplatin treatment when compared with the control cells, whereas the converse results were observed in S26 cells with NEDD8 overexpression. Colony formation assays confirmed that NEDD8-silenced cells were more sensitive to radiation than vector control cells (Figures 3e and f). Together, these data suggest that the inhibition of NEDD8 expression enhances the sensitivity of NPC cells to cisplatin and radiation treatment.

### NEDD8 is involved in the CSC phenotype of NPC cells *in vitro* and *in vivo*

**Figure 4:**
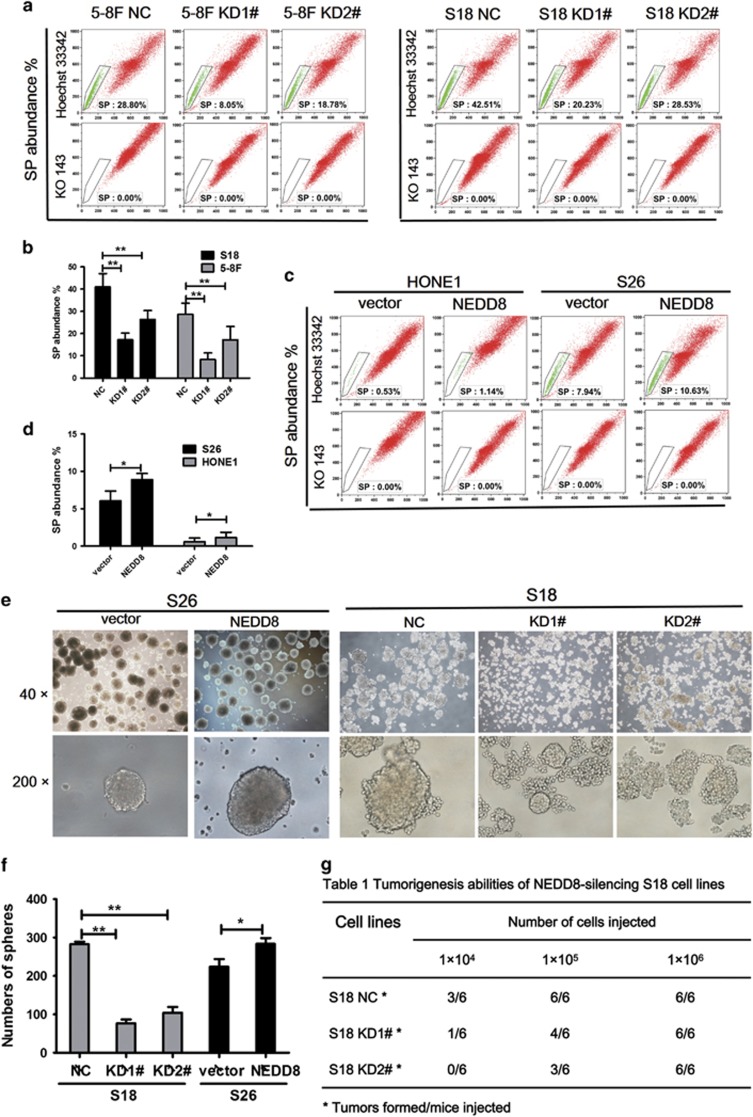
NEDD8 suppresses the stemness of NPC cells *in vitro* and the tumorigenicity *in vivo*. (**a**,**c**) The side population (SP) cells assay. S18 and 5-8 F cells with knockdown NEDD8, S26 and HONE1 with overexpression NEDD8 were treated with 5 *μ*M ko143 and 5 *μ*g/ml Hoechst 33342 dye and tested by flow cytometric analysis. (**b**,**d**) Representative flow cytometric histograms demonstrated a distinct percentages of SP cells. (**e,f**) The images (up panel) and quantifcation of the number of spheres (down panel) formed from the indicated stable cell lines in DMEM/F12 medium with the addition of EGF and FGF for 7–8 days. (**g**) Tumorigenesis abilities: NEDD8-silencing S18 cells in three cell concentrations injected to nude mice and observed for 20 days. The results are presented as the means±S.E.M., and three independent experiments were performed. *P<0.05, **P< 0.01

We further explored the role of NEDD8 in side population (SP) maintenance, as well as in the self-renewal and tumorigenesis of NPC cells. All these aspects have been recognized to be stem cell characteristics. It was found that the population of SP cells in control cells (S18, 5-8F) was ~0.5–3-fold higher than that in NEDD8 KD cells (Figures 4a and b), while in S26 and HONE1 cells, SP rates increased along with NEDD8 overexpression (Figures 4c and d). For the knockdown and overexpression of NEDD8 in S18 and S26 cells, the numbers and sizes of spheres reduced and increased, respectively (Figures 4e and f). The tumorigenesis ability of S18 cells *in vivo* was reduced when NEDD8 was knocked down (Figure 4g). When 1 × 10^6^ cells were injected into nude mice, both the S18-NEDD8 KD1, KD2 cells and the vector control cells developed tumors at a similar rate (6/6). However, when the number of injected cells were reduced to 1 × 10^4^, 50% of the mice (3/6) inoculated with the S18-NC cells formed tumors compared with 17% of the mice (1/6) in the KD1 group, and 0% of the mice (0/6) in the KD2 group. We conclude from these data, that NEDD8 enhances self-renewing properties of CSC in NPC cells.

### Anti-tumor activity of MLN4924 in nasopharyngeal carcinoma

**Figure 5:**
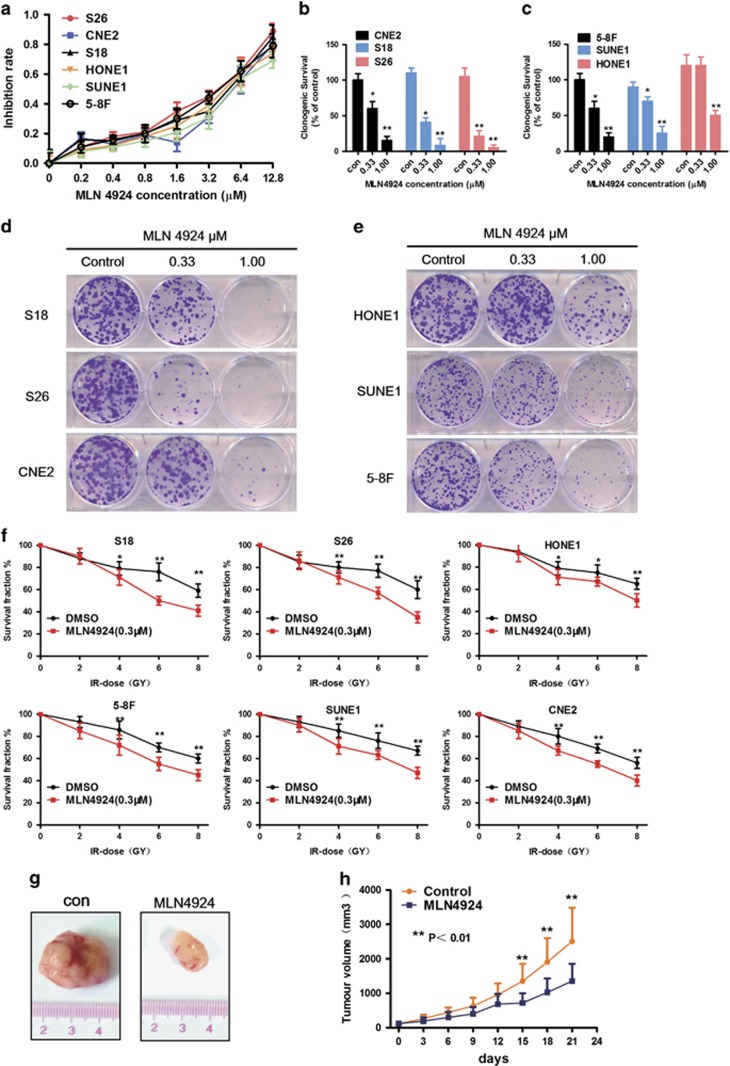
MLN4924 inhibits the growth of human NPC and sensitizes NPC cells to radiation. (**a**) The NPC cells were treated with various concentrations of MLN4924 for 72 h. Cell viability was assessed by MTT assay. mean±S.E.M., *n*=3. (**b,c,d,e**) The colony formation assays were performed in the indicated cell lines for MLN4924. The colonies with more than 50 cells were counted after 10–12 days. (**f**) The ability for colony formation upon various radiation doses combined with MLN4924. Cells were seeded in 60-mm dishes treated with MLN4924 for 6 h and then followed different dose of radiation as indicated. (**g**,**h**) MLN4924 significantly inhibited the growth of human NPC xenografts *in vivo*. Mice bearing S18 human NPC tumor xenografts were treated with HPBCD (as the non-treated control, *n*=9) or MLN4924 (30 mg/kg, *n*=9, subcutaneous injection) twice a day for 10 days. Tumor volumes and mice weights were measured every three day. Data are presented as means ±S.E.M. of three independent experiments. *P<0.05, **P<0.01

The efficacy of MLN4924 in NPC cell lines was further detected in CNE2, SUNE1, S18, S26, 5-8F, and HONE1. In cell viability assays, MLN4924 effectively inhibited the proliferation of cells in a dose-dependent manner and time-dependent manner (Figure 5a). The IC50 of MLN4924 on CNE2 and SUNE1 cells was 3.29 and 3.84 *μ*M, respectively (results are not provided). Likewise, MLN4924 caused a dose-dependent inhibition on colony formation in the NPC cells (Figures 5b–e). NPC cell lines were pretreated with MLN4924 (1 *μ*M) for six hours, followed by radiation at different doses up to 8 GY. The results showed that MLN4924 could sensitize NPC cells to radiation evaluated by colony formation assays (Figure 5f). In order to further confirm the anti-tumor effects *in vivo*, S18 was subcutaneously inoculated to generate xenograft tumors. As shown by the tumor growth curve (Figure 5h), tumors in the MLN4924-treated group grew slowly, while control tumors progressed rapidly. At the end point, subcutaneous xenografts of both groups were collected and imaged (Figure 5g). As shown in Figure 5g, the size of tumors in the MLN4924-treated group was significantly smaller than in the control group. During treatment, no obvious treatment-related toxicity was observed. Thus, these data clearly indicate that MLN4924 is a potent anti-tumor compound against NPC.

### MLN4924 induces apoptosis and alters cell cycle progression

**Figure 6:**
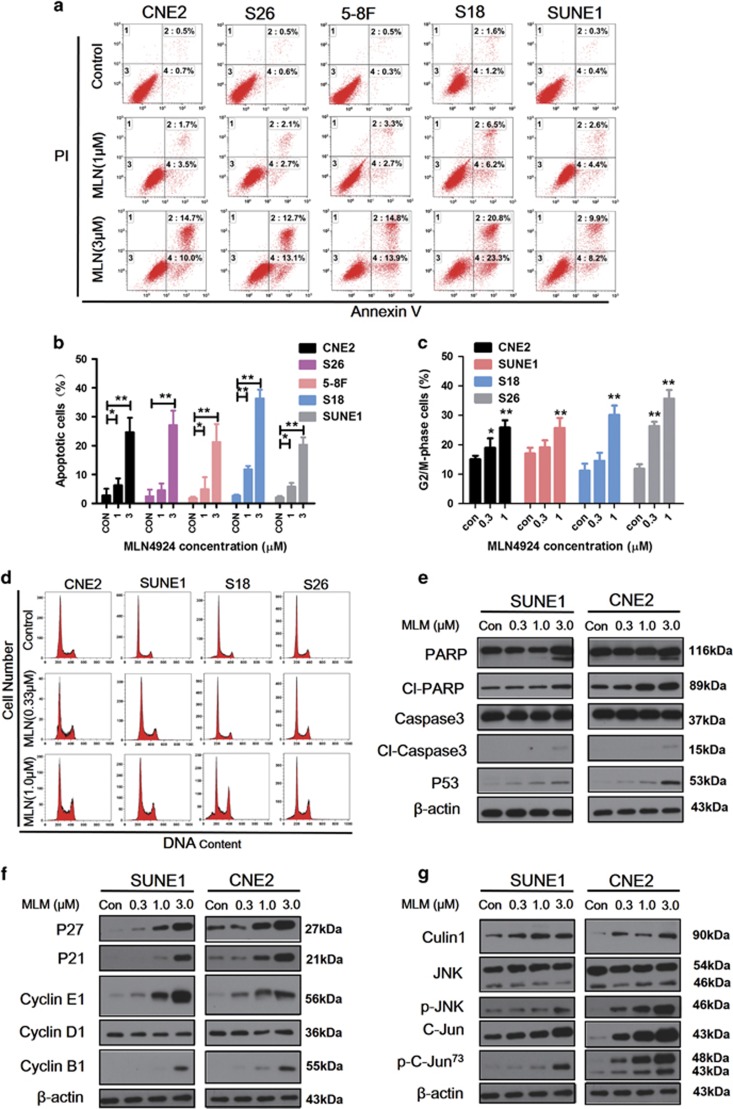
Effects of MLN4924 on apoptosis and cell cycle progression in human NPC cell lines. (**a**,**b**) CNE2, S18, S26, 5-8Fand SUNE1 were treated with MLN4924 at 1 *μ*M and 3 *μ*M for 48 h, followed by FACS for apoptosis analysis. (**c**,**d**) The given cell lines were exposed to the indicated concentrations of MLN4924 48 h and then harvested for cell cycle profiling. (**e–g**) CNE2 and SUNE1 were lysed after MLN4924 treatment for 48 h, and western blot analysis to evaluate the expression of protein and *β*-actin as a loading control. Data are presented as means ±S.E.M. of three independent experiments. *P<0.05, **P<0.01 as compared with the controls

Flow cytometry was used to evaluate the effect of MLN4924 on apoptosis and cell cycle distribution. Results revealed that MLN4924-induced apoptosis in CNE2, SUNE1, S18, S26, and 5-8 F cells (Figures 6a and b). Cell apoptosis rates were increased in a dose-dependent manner from 2.8% up to 24.7% in CNE2, from 2.5% up to 27.1% in S26, from 1.9% up to 21.3% in 5-8 F, from 2.7% up to 36.4% in S18, and from 2.1% up to 20.4% in SUNE1.The effect of G2/M arrest was observed48 h after MLN4924 treatment, again in a dose-dependent manner (Figures 6c and d). MLN4924 treatment caused the deneddylation of cullin1 (Figure 6g). The accumulation of cleaved PARP and caspase-3, as well as p53, were observed (Figure 6e). These obviously increased in cell cycle inhibitors p21, p27, cyclinE1, and cyclinB1; but there was no change in the cyclin D1 level (Figure 6f). MLN4924 significantly enhanced c-Jun expression, as well as the phosphorylation of c-Jun (Figure 6g).

**Figure 7:**
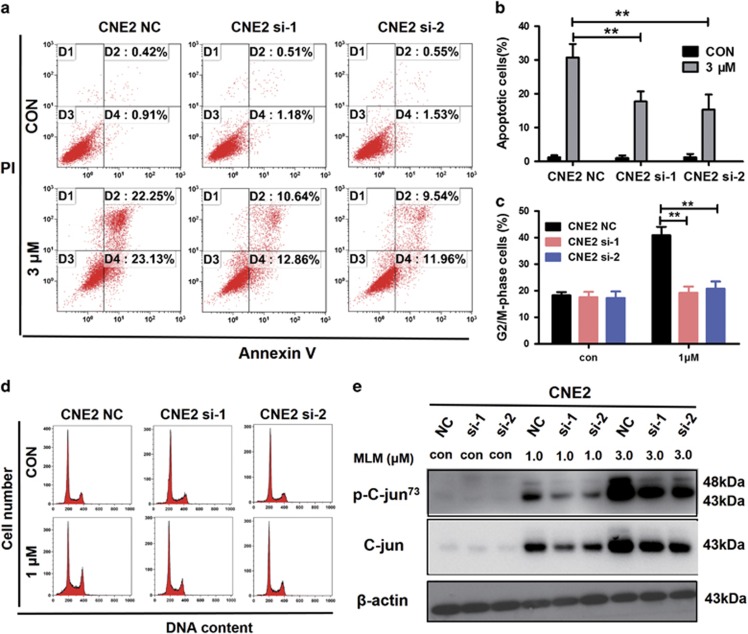
C-Jun knockdown inhibits cell apoptosis and cell cycle arrest in human NPC cell. (**a**,**b**) CNE2 (NC, si-1, si-2) were treated with MLN4924 at 3 *μ*M for 48 h after c-Jun was transiently knocked down using siRNA (si-1 and si-2), followed by apoptosis analysis. (**c**,**d**) Cell cycle analysis was tested after CNE2 (NC, si-1, si-2) exposed to 1 *μ*M MLN4924. (**e**) Western blot analysis to evaluate the expression of c-Jun, p-c-Jun, and *β*-actin as a loading control. Data are presented as means ±S.E.M. of three independent experiments. *P<0.05, **P<0.01 as compared with the controls

The gene-silencing efficacy for c-Jun was evaluated 48 h after transfection using real-time PCR and immunoblotting (Supplementary Figures S1a and b). For cell proliferation and the colony formation assays, the cells were seeded in cell plates after post-transfection for 48 h. There were no differences in cell proliferation (Supplementary Figure 1e) or the colony formation (Supplementary Figures S1c and d) between c-Jun si-1, si-2, and c-Jun NC cells. Transient interfering c-Jun expression in CNE2 cell, MLN4924-induced apoptosis and cell cycle arrest were rescued. The apoptosis rate in the NC group was markedly higher than in si-1 and si-2 groups (Figures 7a and b). G2/M arrest almost decreased 50% (from 40% to 19%) (Figures 7c and d). The MLN4924-induced accumulation of total c-Jun protein as well as the phosphorylated c-Jun could be suppressed by transient knocking down of the gene JUN (Figure 7e). Taken together, these results reveal that MLN4924 could induce apoptosis and alter cell cycle progression, and these effects might be regulated through c-Jun neddylation.

### MLN4924 decreases the SP subpopulation and inhibits the ABCG2− cells and ABCG2+ cells growth

**Figure 8:**
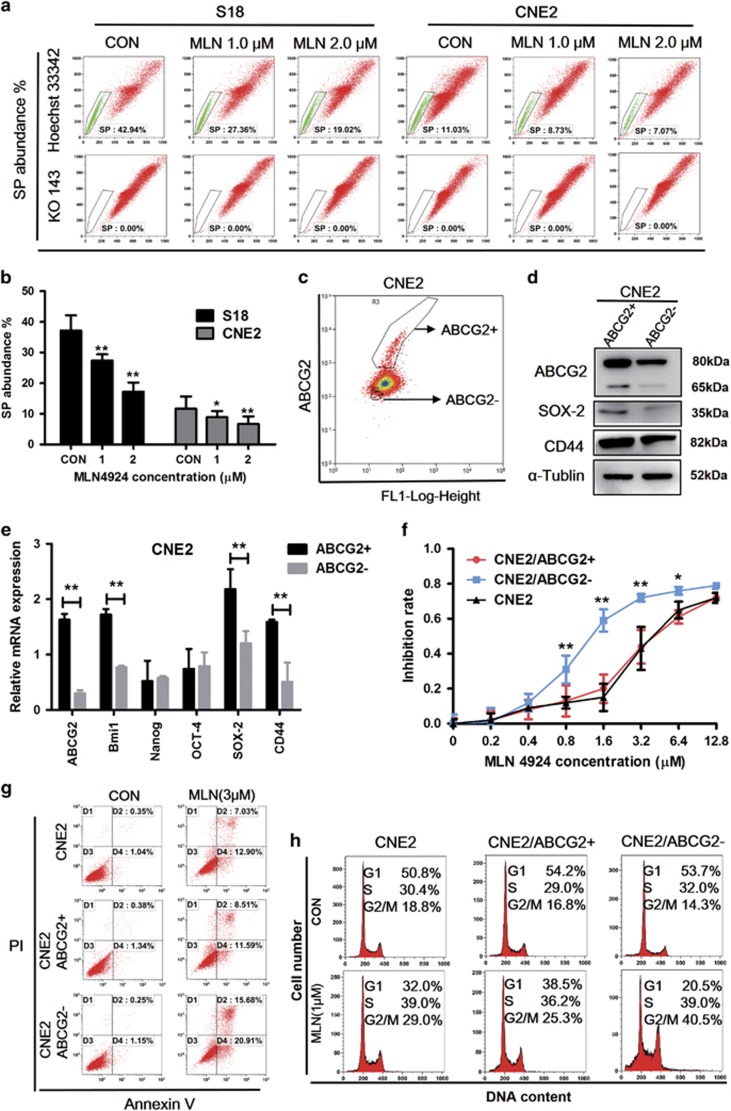
MLN4924 attenuates SP rate and inhibits the CSCs and the non CSCs of human NPC cells. (**a**,**b**) The SP assay for S18 and CNE2 subjected with MLN4924 in the indicated concentration for 48 h. (**c**) CNE2 was treated by ABCG2 antibody using FACS; ~5% of the ABCG2+/High and ABCG2-/Low cells were sorted. (**d**,**e**) ABCG2+/High and ABCG2-/Low cells were tested the expression of ABCG2, CD44, SOX-2, OCT-4, Bmi1, Nanog using mRNA, and WB. (**f**) CNE2, CNE2/ABCG2+ and CNE2/ABCG2− cells were treated with various concentrations of MLN4924 for 72 h after sorting and cultivated for one generation. Cell viability was assessed by MTT assay. (**g**) The indicated cells were treated with MLN4924 (0, 3 *μ*M) for 48 h and test for apoptosis. (**h**) CNE2, CNE2/ABCG2+ and CNE2/ABCG2− cells were harvested for cell cycle analysis after MLN4924 treatment for 48 h. Data are presented as means ±S.E.M. of three independent experiments. *P<0.05, **P<0.01

Treatment with MLN4924 decreased the proportion of SP cells in S18 and CNE2 cells in a dose-dependent manner (Figures 8a and b). The percentage of SP cells decreased from 37.14% in the control group to 17.2% in the treated group (MLN4924, 2 *μ*M). The same effect was observed in the CNE2 cell line. Approximately 5% of the ABCG2− and the ABCG2+ cells were sorted from CNE2 cell line using FACS (Figure 8c) and cultivated one generation for cell experiments. The mRNA and protein expression levels of the CSC markers ABCG2, Bmi1, CD44, SOX-2 were higher in the ABCG2+ cells than in the ABCG2− cells (Figures 8d and e). We used MTT and FACS to test MLN4924’s effect on those cells. MLN4924 could inhibit CNE2, CNE2/ABCG2− and CNE2/ABCG2+ cell proliferation, and induce apoptosis and G2/M phase arrest. CNE2/ABCG2- cells were more sensitive to MLN4924 than CNE2, CNE2/ABCG2+ cells. These data suggest that MLN4924 could kill both CSC cells and non-CSC cells in NPC.

## Discussion

In this study, we confirmed that NEDD8 could be a novel oncogene, and MLN4924 is a promising therapeutic agent for NPC. The elevated expression of NEDD8 in NPC tissues was associated with poorer prognosis in patients with NPC. Our functional studies revealed that knockdown of NEDD8 expression suppressed cancer cell proliferation, colony formation and NPC cell stemness characteristics such as self-renewal, tumorigenesis, radiation, and drug resistance. MLN4924 inhibited NPC cell growth *in vitro* and suppressed the growth of human NPC xenografts *in vivo*. MLN4924 treatment promoted apoptosis and cell cycle arrest via suppressing c-Jun degradation. In addition, MLN4924 reduced the SP percentage and sensitized NPC cells to radiotherapy. This report reveals that NEDD8 promotes tumorigenesis in NPC and therefore, serves as a potential therapeutic target.

The therapeutic value of regulating the viability of the ubiquitin–proteasome pathway in cancer has been proven by the proteasome inhibitor bortezomib (Velcade), which has been applied to multiple myeloma in clinic.^27^ Thus more efforts are needed to search for further potential targets in this pathway. Protein neddylation is a post-translational modification that adds the ubiquitin-like molecule NEDD8 to substrate proteins.^12, 13^ Neddylation regulates the degradation of numerous proteins, while its dysfunction leads to carcinogenesis.^28, 29^ Some recent studies have reported that the neddylation pathway is overactivated in several types of human cancers, and inhibition of this pathway significantly suppresses tumor growth.^16, 17, 18, 21, 22, 30^ In both lung adenocarcinoma and squamous-cell carcinoma, the neddylation pathway was upregulated.^30^ This consistent phenomenon was observed in the NPC in our study. Our findings confirm that the over-activity of the neddylation pathway in human cancer is a suitable condition for applying MLN4924 a specific NAE inhibitor to deter CRL neddylation. This drug inactivates CRL/SCF E3 ligase and results in the accumulation of a large amount of its substrates, leading to the suppression of tumor growth.^20^ Triggering cell cycle disturbance, apoptosis, autophagy and senescence have also been reported to be responsible for the anti-tumor effects of MLN4924,^18, 20, 31^ as well as the inhibition of tumor angiogenesis and metastasis.^22, 30^ Our findings were not only consistent with the findings of other studies, but also reveal that MLN4924 induces G2/M arrest and apoptosis *via* the regulation of c-Jun degradation. c-Jun is a well-known substrate of SAG-SCF E3 ligase.^32^

It has been claimed that CSCs are responsible for metastasis and treatment resistance in NPC, inevitably resulting in treatment failure.^4, 33^ Interestingly, the silencing of endogenous NEDD8 dramatically represses NPC stem-like features, as observed in the SP assay, spheroid formation assay and tumorigenesis, and enhances cisplatin and radiation efficacy in killing cancer cells. Moreover, MLN4924 reduces the percentage of SP cells in NPC cells in a dose-dependent manner, while the population of SP cells in cisplatin treated S18 cells reached up to 90%.^34^ Wang’s study found that the SP assay was a viable method to identify cancer stem cell-like cells in human NPC cell lines.^35^ SP assay is based on the ATP-binding cassette (ABC) half transporter member 2 of G family protein (ABCG2), which can efflux Hoechst 33342 out of cells. The PE-cy5.5 conjugated anti-ABCG2 antibody was used to sort ABCG2− (less cancer stem cell-like) and ABCG2+ (more cancer stem cell-like) populations. Our study finds that MLN4924 can kill ABCG2− cells and ABCG2+ cells, thus having a great potential for clinical application.

In summary, we have determined that NEDD8 has an important role in the tumorigenesis of NPC and could serve as a useful biomarker for improving the prediction of NPC patient prognosis. Moreover, using MLN4924 in combination with chemotherapy and radiotherapy may provide a promising new avenue for NPC therapy.

## Materials and Methods

### Reagents and cell lines

MLN4924 was a gift from Fudan University. For *in vitro* studies, MLN4924 was dissolved in dimethyl sulfoxide (DMSO) and kept in −20 °C. MLN4924 was dissolved in 10% 2-hydroxypropyl- b-cyclodextrin (HPBCD) for the *in vivo* studies. The MLN4924 solution was freshly made every week and stored in a dark at room temperature before use.

Cell culture reagents and fetal bovine serum (FBS) were obtained from Gibico (Grand Island, NY, USA). The Oligofectamine reagent and Western Lightning Chemiluminescence Plus reagent was from Thermo Scientific Pierce (Waltham, MA, USA). The Annexin-V/propidiumiodide (PI) kit was from BD Biosciences Franklin Lakes, NJ, USA). Human NPC cells used in this study were stored in our lab and were cultured in DMEM medium containing 10% FBS at 37 °C in 5% CO_2_.

### Cell proliferation assays and cell viability assays

For the proliferation assay, CNE2, SUNE1, S18, S26, 5-8 F, HONE1 cells were seeded at a density of 1 × 10^3^ cells per well in 96-well plates and cultured for six days and tested MTT every day. For cell viability analysis, the density of cells was 2 × 10^3^ cells per well in 96-well plates. A day after seeding, cells were stained with a different concentration of MLN4924 and seeded for 72 h. At the end point, cells were given 20 *μ*L MTT dye (0.5 mg/ml, Sigma, Shanghai, China) for 4 h at 37 °C, then the culture medium was removed, and the cells dissolved in DMSO. The absorbance was measured with a multifunctional plate reader. Each experiment was performed three times.

### Colony formation assay

Cells were seeded in triplicate in 6-well culture plates at a density of 500 cells/well. For the radiotherapy and drug therapy, cells were irradiated with different GY radials and stained the following day with a different concentration of MLN4924, respectively. The culture medium was subsequently changed every three days. After 11–13 days, the resulting colonies were fixed with methanol and stained with 0.2% crystal violet. Colonies that contained greater than 50 cells were counted. All experiments were independently repeated at least three times.

### Measurement of cell cycle and apoptosis

Cells from each cell line were grown in the medium mentioned above. At 50% confluency, the cells were treated with DMSO (as the non-treated control) or MLN4924 (0.33, 1.0 *μ*M) for 48 h. Then, the cells were collected and processed with a cell cycle staining kit (MultiSciences, Hangzhou, China) for cell cycle analysis. Cell cycle distribution was analyzed using a FACS flow cytometer (BD Bioscience). For the apoptosis assay, the cells were harvested after treatment with different doses of MLN4924 or cisplatin for 48 h. Cells were stained with an Annexin-V-FITC apoptosis detection kit (BD Bioscience), and apoptotic cells were identified and quantified using flow cytometry, according to the manufacturer’s instructions.

### Quantitative real-time PCR

The expression levels of gene were determined by real-time RT-PCR. Total RNA from NPC cells were extracted using Trizol reagent (Invitrogen, CA, USA). Beta-actin served as the normalization genes for these studies. The relative expression levels of the target genes were calculated as two power values of ΔCt (the Ct of GAPDH minus the Ct of the target gene). The PCR primer sequences are as follows:

NEDD8-forward primer 5′–3′CGCTGACCGGAAAGGAGATT

NEDD8-reverse primer 5′–3′CAGAGCCAACACCAGGTGAA

CD44-forward primer 5′–3′TCCATCAAAGGCATTGGGCAG

CD44-reverse primer 5′–3′AACCTGCCGCTTTGCAGGTGT

Bmi1-forward primer 5′–3′TGGAGAAGGAATGGTCCACTTC

Bmi1-reverse primer 5′–3′GTGAGGAAACTGTGGATGAGGA

SOX-2-forward primer 5′–3′AAATGGGAGGGGTGCAAAAGAGGAG

SOX-2-reverse primer 5′–3′CAGCTGTCATTTGCTGTGGGTGATG

Nanog-forward primer 5′–3′AATACCTCAGCCTCCAGCAGATG

Nanog-reverse primer 5′–3′TGCGTCACACCATTGCTATTCTTC

ABCG2-forward primer 5′–3′TCATCAGCCTCGATATTCCATCT

ABCG2-reverse primer 5′–3′GGCCCGTGGAACATAAGTCTT.

### Sphere culture

For the tumor sphere assay, the cells were seeded on 6-well culture plates (low-attachment, corning, NY, USA) at a density of 4000 cells/well. Cells were grown in serum-free DMEMF/12 medium supplemented with 20 ng/ml of epidermal growth factor (EGF) and 20 ng/ml of basic fibroblast growth factor (bFGF). The number of spheres with a diameter of >50 *μ*m was quantified by Image J software (National institute of Health, Bethesda, MD, USA). Three independent experiments were performed.

### Side population analysis

After treatment with different doses of MLN4924 (0, 1.0, 2.0 *μ*M) for 48 h, the cells were harvested. Stable transfected cells were collected when cells were at a growth of 80%. For the side population analysis, cells were incubated with Hoechst 33342 (5 mg/ml), with or without ko143 (5 *μ*M, Sigma), and incubated in the dark for 90 min at 37 °C with intermittent mixing and tested by flow cytometry.

### Cell sorting

For cell sorting experiments, the PE-cy5.5 conjugated anti-ABCG2 antibody (BD Bioscience, 561460) was used. The labeled CNE2 cells were sorted using FACS. For the ABCG2- and ABCG2+ populations, only 5% of the most lightly stained and the most brightly stained cells were selected. Cells were collected and cultivated for cell experiments.

### Immunoblotting analysis

Western blotting was performed according to standard methods as previously described.^6^ The primary antibodies also included NEDD8 (CST, Danvers, MA, USA, 2754), PARP (CST, 9532), cleaved PARP (CST, 6525), caspase-3 (CST, 9662), P53 (Abcam, Cambridge, UK, ab32049), P21 (CST, 2947), P27 (CST, 3686), cyclin D1 (CST, 4656), cyclinB1 (CST, 12231), cyclinE1 (abcam, ab3927), SAPK/JNK (CST, 9258), phosphor-SAPK/JNK (CST, Thr183/Tyr185, 4668), c-Jun (CST, 60A8), phospho-c-Jun (CST, Ser63,9261), phospho-c-Jun (CST, Ser73,3270), *β*-actin (proteintech, Wuhan, China, 60008-1-Ig), GAPDH (proteintech, 60004-1-Ig), alpha Tubulin (proteintech, 66031-1-Ig), ABCG2 (proteintech, 10051-1-AP). Anti-mouse and anti-rabbit peroxidase conjugated secondary antibodies were purchased from Proteintech.

### Stable cell lines

In order to establish stable cell lines with the NEDD8 knockdown, we selected two effective shRNA sequences. The following are primers of NEDD8 shRNA: shRNA-1: 5′-ggcatcatatatcctctcact-3′ shRNA-2: 5′-gcggtaggagcagcaatttat-3′. The 293T cells were co-transfected with expression vectors and virus skeleton vectors using X-tremeGENE HP DNA transfection reagent (Roche, Mannheim, Germany). Infectious lentiviruses were collected after transfection for 48 h, and filtered by a 0.45-*μ*m filter (Millipore, Boston, MA, USA). Then, the lentiviruses were used to infect cells. The stable cell lines were selected with 1 *μ*g/ml of puromycin (Sigma) for three days.

### Small interfering RNA transfection

Transient transfections were performed using the Lipofectamine RNAiMAX Reagent (Invitrogen) according to the manufacturer’s instruction. The siRNAs and transfection reagent diluted in Opti-MEM Medium (Invitrogen) were mixed and incubated at room temperature for 15 min, and added to the cells. Small interfering RNAs were purchased from ribobio, and siRNA targeting human c-Jun are 5′- CCAAGAACGTGACAGATGA-3′ (si#1) and 5’-CGCAGCAGTTGCAAACATT-3′ (si#2).

### Animal experiments

All animal experiments were approved by the Sun Yat-Sen University Cancer Center Institutional Animal Care and Usage Committee. Five-week-old female BALB/c (nu/nu) nude mice were obtained from the Shanghai experimental animal center (Shanghai, China). Mice were housed under standard conditions. All procedures were performed in accordance with the National Institutes of Health Guide for the Care and Use of Laboratory Animals.

For the tumorigenesis assay, BALB/c (nu/nu) nude mice were randomly divided into nine groups (*n*=6 for each group). S18 cells transfected with NEDD8 shRNA (KD1# and KD2#) or negative control shRNA (NC) cells (three cellular concentrations: 1 × 10^6^, 1 × 10^5^, and 1 × 10^4^) were suspended in 50 *μ*l of DMEM medium. Cells were mixed with matrigel (50 *μ*l) and subcutaneously injected into the flank of each mouse. Tumor diameters were measured, and the volume (length × width^2^ × 0.5236) was calculated every three days.

For the compound effect assay, S18 cells were re-suspended in PBS and subcutaneously injected into the right flank with 5 × 10^6^ cells per 100 *μ*l. One week later, tumor bearing mice were randomly divided into two groups (9 mice/group); and treatment with 10% HPBCD (control) or MLN4924 (30 mg/kg) by subcutaneous injection twice a day, respectively, on a 5-days-on/5-days-off schedule for two cycles within a total of 21 days. The size of the tumors was measured using a vernier caliper every three days. At the end of the study, the mice were euthanized; and tumor tissues collected, photographed, and weighed.

### Human tissue specimens

A total of 197 paraffin-embedded primary NPC tissues, diagnosed between 2005 and 2013, were retrieved from the Department of Pathology, Sun Yat-Sen University Cancer Center (SYSUCC), with the approval of the Institutional Clinical Ethics Review Board at SYSUCC. Median follow-up time for all patients was 68 months. All human tissue samples were provided following patient consents. The IHC scores for NEDD8 in NPC tissues were calculated by two independent pathologists. This consisted of a score for the percentage of positively stained tumor cells and the grade of staining intensity. The intensity score represented the cytoplasmic and nucleus staining intensity of positive cells. The staining intensity was divided as follows: no staining=0, weak staining=1, moderate staining=2 and strong staining=3. The percentages of positive cells were categorized as follows: no staining=0, 1–10% of stained cells=1, 11–50%=2, 51–80%=3 and 81–100%=4. Then, the proportion and intensity were multiplied to produce a total score of 0 through 12.

### Statistical analysis

Statistical analyses were performed using SPSS version 21.0 for Windows (IBM, NY, USA). The median IHC staining score was used as a cut-off value to divide the patients into low and high NEDD8 expression groups. The correlations between the NEDD8 expression and OS and DFS were analyzed with Kaplan–Meier survival and the log-rank test. Chi-squared tests were used to analyze the relationship between the NEDD8 expression and clinicopathological status. The significance of several variables for survival were analyzed using the Cox regression model in the multivariate analysis stage. Data were analyzed using Student’s *t*-test or one/two way ANOVA methods and represented as the means±S.E.M.; *P*<0.05 was considered statistically significant.

## Figures and Tables

**Table 1:** Association of NEDD8 expression and patient clinicopathological characteristics in nasopharyngeal carcinoma tissues

**Characteristics**	**Number**	**NEDD8 expression level**		***P*-value (***χ*^2^ **test)**
		**Low**	**High**	
*Gender*				
Male	149	76	73	0.572
Female	48	27	21	

*Age*				
⩽45	109	57	52	0.998
>45	88	46	42	

*T stage*				
T1–3	147	78	69	0.708
T4	50	25	25	

*N stage*				
N0–1	115	68	47	**0.023**
N2–3	82	35	47	

*M stage*				
M0	186	99	87	0.357
M1	11	4	7	

*Clinical staging*				
I–II	120	64	56	0.713
III–IV	77	39	38	

*Death*				
No	163	93	70	**0.003**
Yes	34	10	24	

*Disease progression*				
No	172	94	78	0.081
Yes	25	9	16	

Bold values indicate statistically significant values.

**Table 2:** Univariate and multivariate analysis for overall survival in NPC patients (Cox proportional hazards regression model)

**Variables**	**Univariate analysis**			**Multivariate analysis**		
	**HR**	**CI**	***P*-value**	**HR**	**CI**	***P*-value**
Age: ⩽45 *versus* >45	0.213	0.096–0.472	**0.001**	0.206	0.093–0.458	**0.001**
Gender: male *versus* female	1.428	0.585–3.486	0.434			NS
T stage: T1–3 *versus* T4	1.066	0.338–3.367	0.913			NS
N stage: N0–1 *versus* N2–3	0.782	0.356–1.718	0.541			NS
M stage: M0 *versus* M1	0.176	0.049–0.629	**0.007**	0.156	0.054–0.451	**0.001**
Clinical stage: I–II *versus* III–IV	0.527	0.179–1.553	0.247	0.529	0.252–1.113	0.093
NEDD8 expression: low *versus* high	0.315	0.148–0.668	**0.003**	0.303	0.144–0.640	**0.002**

Abbreviations: HR, hazard ratio; CI, confidence interval.

*P*-values were calculated by log-rank test. Bold values indicate statistically significant values.
