# Supplementary figures and images for: Promoting tumorigenesis in nasopharyngeal carcinoma, NEDD8 serves as a potential theranostic target
Source: Cell Death Dis. 2017 Jun 1;8(6):e2834–. doi: 10.1038/cddis.2017.195 (PMC5520881; doi:10.1038/cddis.2017.195)

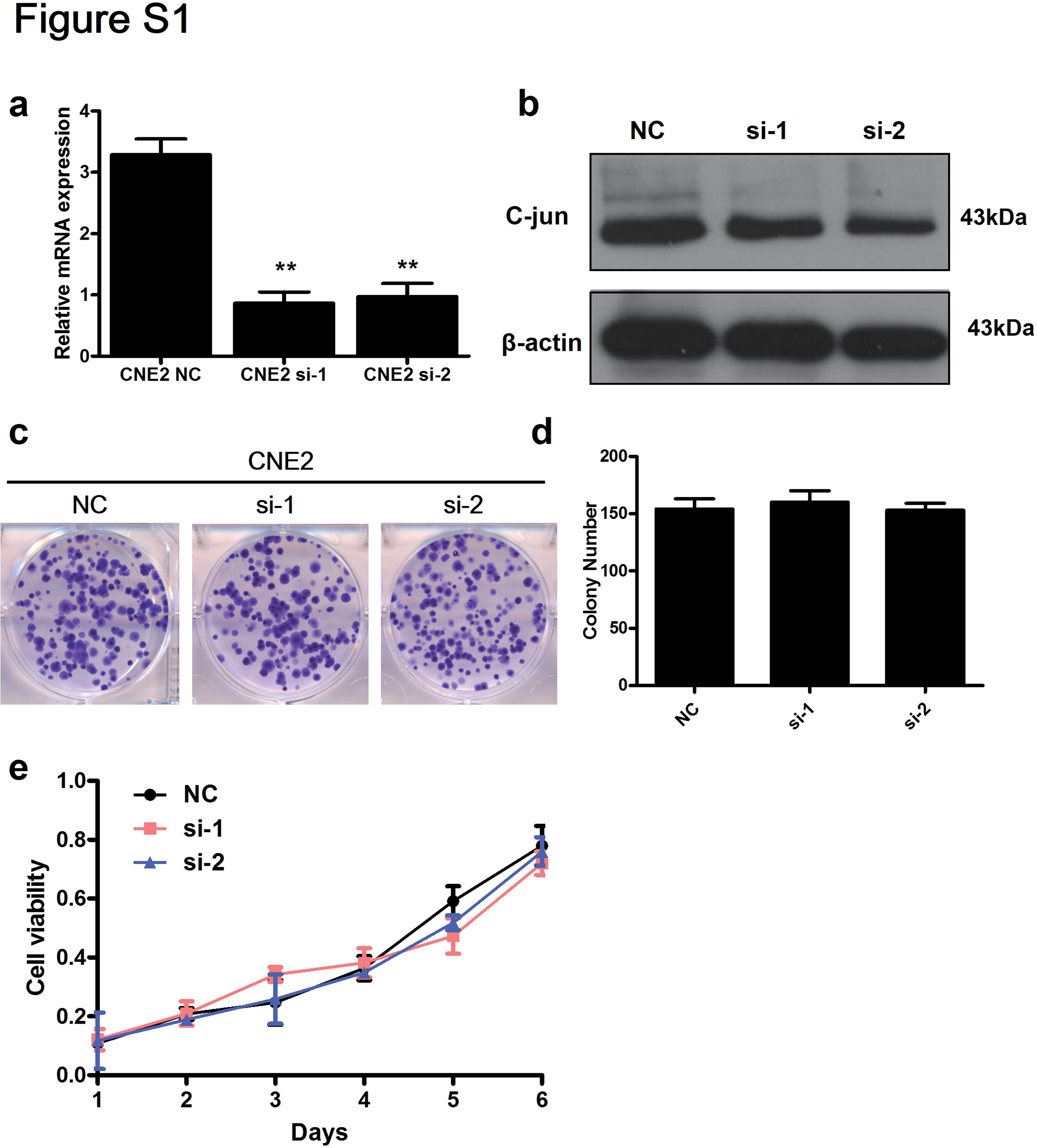

Supplement: Supplementary Figure S1 [file cddis2017195x1.tif]
