# Supplementary material for: Promoting tumorigenesis in nasopharyngeal carcinoma, NEDD8 serves as a potential theranostic target
Source: Cell Death Dis. 2017 Jun 1;8(6):e2834–. doi: 10.1038/cddis.2017.195 (PMC5520881; doi:10.1038/cddis.2017.195)
Supplement: Supplementary Figure S1 Legend [file cddis2017195x2.docx]

**Supplementary Figure legends**

**Figure S1 Suppression of c-Jun expression not affects growth in NPC cells *in vitro.***

**(a)** C-Jun was depleted in CNE2 cell by RNAi and tested by RT-PCR. **(b)** Western blotting analysis of c-Jun expression; β-actin was used as a loading control. **(c, d)** The colony-formation ability of c-Jun-depleted CNE2 cells not increased compared with NC control cells. **(e)** c-Jun depletion not affected NPC cell proliferation compared with NC control cells as determined by MTT assay. Three independent experiments were performed; *P< 0.05, **P< 0.01 vs NC control cells.
